# Structural Modeling and DNA Binding Autoinhibition Analysis of Ergp55, a Critical Transcription Factor in Prostate Cancer

**DOI:** 10.1371/journal.pone.0039850

**Published:** 2012-06-28

**Authors:** Shanti P. Gangwar, Sharmistha Dey, Ajay K. Saxena

**Affiliations:** 1 Structural Biology Lab, School of Life Sciences, Jawaharlal Nehru University, New Delhi, India; 2 Department of Biophysics, All India Institutes of Medical Sciences, New Delhi, India; University of South Florida College of Medicine, United States of America

## Abstract

**Background:**

The Ergp55 protein belongs to Ets family of transcription factor. The Ets proteins are highly conserved in their DNA binding domain and involved in various development processes and regulation of cancer metabolism. To study the structure and DNA binding autoinhibition mechanism of Ergp55 protein, we have produced full length and smaller polypeptides of Ergp55 protein in *E. coli* and characterized using various biophysical techniques.

**Results:**

The Ergp55 polypeptides contain large amount of α-helix and random coil structures as measured by circular dichorism spectroscopy. The full length Ergp55 forms a flexible and elongated molecule as revealed by molecular modeling, dynamics simulation and structural prediction algorithms. The binding analyses of Ergp55 polypeptides with target DNA sequences of E74 and cfos promoters indicate that longer fragments of Ergp55 (beyond the Ets domain) showed the evidence of auto-inhibition. This study also revealed the parts of Ergp55 protein that mediate auto-inhibition.

**Significance:**

The current study will aid in designing the compounds that stabilize the inhibited form of Ergp55 and inhibit its binding to promoter DNA. It will contribute in the development of drugs targeting Ergp55 for the prostate cancer treatment.

## Introduction

The Ets family proteins share highly conserved winged helix-turn-helix DNA-binding domain and bind to consensus DNA core sequence 5′-GGA (A/T)-3′ [1]. The Erg proteins belong to Ets family of transcription factor. Erg gene is rearranged in human myeloid leukemia [2] and in 5–10% of patients with Ewing's sarcoma [3]. In both cases, chromosomal translocations results in the expression of oncogenic fusion proteins composed of Erg and member of Tet subfamily of RNA binding proteins. Erg protein is essential for definitive hematopoiesis, adult hematopoietic stem cell function and maintenance of normal peripheral blood platelet numbers [4]. The TMPRSS2-Erg fusion oncogene transcripts observed in prostate cancer cells are significantly associated with aggressive cancer, metastatic spread and increased probability of death [5].

The Erg gene encodes five proteins, Erg-1, Erg-2, Ergp55, Ergp49 and Ergp38 as a result of different splicing, polyadenylation or initiation codon. The Ergp55 isoform contains four functional domains, which are involved in DNA binding, transcriptional activation and negative regulation of transactivation [6]. The Ergp55 protein forms dimer with itself and with two other isoforms Ergp49 and Ergp38, via PNT and Ets domain [7]. The central domain of Ergp55 behaves as inhibitory domain on dimerization and its removal enhances the transactivation property (7). The critical residues of Ets domain of Ergp55, which mediate Ergp55-jun/fos-DNA ternary complex formation, have been identified and characterized [8].

So far, tertiary structure of any full-length Ets protein is not determined. However, structures of DNA-binding domains of several Ets proteins have been determined using X-ray crystallography and nuclear magnetic resonance techniques [9]–[16]. The gonome-wide analysis of Ets-family DNA-binding *in vitro* and *in vivo* has been studied recently [17].

The precise mechanism by which, Ergp55 protein acts on transcription is not understood. To understand the structure and DNA binding autoinhibition mechanism of Ergp55, we performed circular dichorism, molecular modeling and theoretical structural prediction analysis on Ergp55 polypeptides. To understand the DNA binding autoinhibition mechanism, the binding studies of Ergp55 polypeptides with DNA sequences of E74 and cfos promoters were carried out. Our results indicated that (i) Ergp55 polypeptides contains high percentage of α-helix and random coil structures (ii) full length Ergp55 is a flexible and elongated molecule (iii) longer fragments beyond the canonical Ets domain of Ergp55 showed the evidence of autoinhibition.

## Materials and Methods

### Construction of Ergp55 plasmids

The full length Erg_1–479_ and Erg_112–399_ genes were cloned in pET28a (+) vector. The Erg_1–399_ and Erg_307–399_ genes were cloned in pRSETA and pET21a (+) expression vector. All deletion mutant genes were amplified from full-length Erg_1–479_ plasmid using polymerase chain reaction.

**Figure 1:**
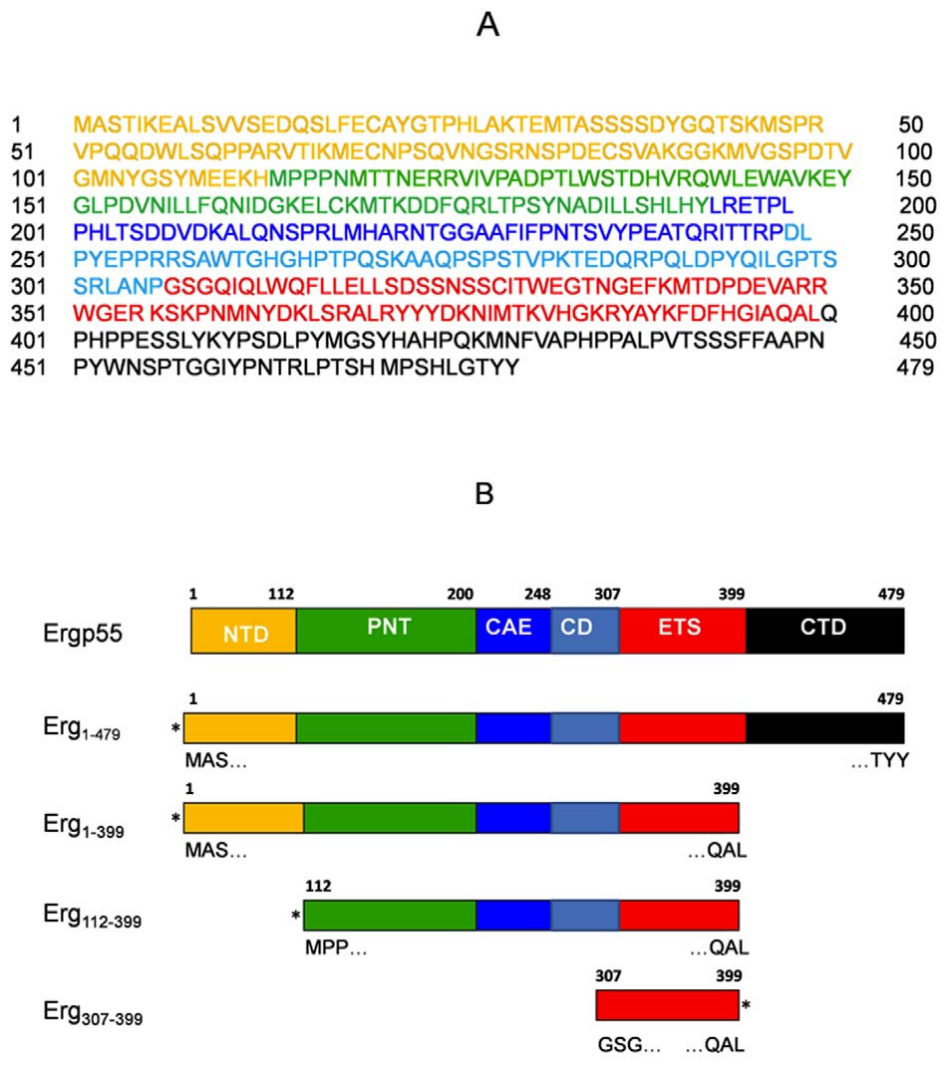
The human Ergp55 protein sequence. (A) Shown here the amino acid sequences full length Ergp55 without 6xHis tag and cleavage site. The residues were highlighted according to the size of Ergp55 domains, NTD (yellow), PNT (green), CAE/CD (blue/cyan), Ets (red) and CTD (black). (B) Shown here the Ergp55 constructs used in the experiments (coloring scheme same as in Fig. 1A). The labeled residues defining the sequence of beginning and end of constructs. *Aestericks* denote the position of 6xHis tag on various Ergp55 constructs.

### Purification of Ergp55 polypeptides

The Erg_1–479,_ Erg_1–399_, Erg_112–399_ and Erg_307–399_ plasmids were transformed into *E. coli*. BL21 (DE3) cells. The cells were grown in 3 liters luria bertani media containing appropriate antibiotics at 37°C, until OD_600_ reached to 0.6. The 0.125 mM IPTG was induced at 37°C and culture further shaked for 4 h at 220 rpm. The cells were harvested by centrifugation at 6000x *g* for 10°min at 4°C. The cells were suspended in 50 ml of lysis buffer containing (25 mM Tris-HCl pH 8.0, 300 mM NaCl, 1 mM benzamidine-HCl, 0.1% triton X-100, 5% glycerol, 3 mM 2-mercaptoethanol, 1 mM phenylmethylsulfonyl fluoride and 0.5 mg/ml lysozyme) and disrupted by sonication at 4°C. The crude lysate was centrifuged at 25000× *g* for 20 min at 4°C. The supernatant was loaded on Ni-NTA column, pre-equilibrated with binding buffer (25 mM Tris-HCl pH 8.0, 300 mM NaCl, 1 mM benzamidine-HCl, 5% glycerol, 2 mM 2-mercaptoethanol, 1 mM phenylmethylsulfonyl fluoride and 10 mM imidazole). The proteins were eluted from column with elution buffer (25 mM Tris-HCl pH 8.0, 300 mM NaCl, 1 mM benzamidine-HCl, 5% glycerol, 3 mM 2-mercaptoethanol, 1 mM phenylmethylsulfonyl fluoride and 250 mM imidazole). The eluted fractions were pooled, concentrated and loaded on Sephacryl S-200 HR gel-filtration column pre-equilibrated in buffer (25 mM Tris-HCl pH 8.0, 150 mM NaCl, 3 mM 2-mercaptoethanol and 5% glycerol). The purified proteins were concentrated to 10 mg/ml using amicon ultra centrifugal filter device (Mw cutoff∼ 10 kD). The protein concentration was measured by UV radiation at 280 nm by using extinction coefficient calculated with EXPASY software (http://us.expasy.org/tools/protparam.html).

**Figure 2:**
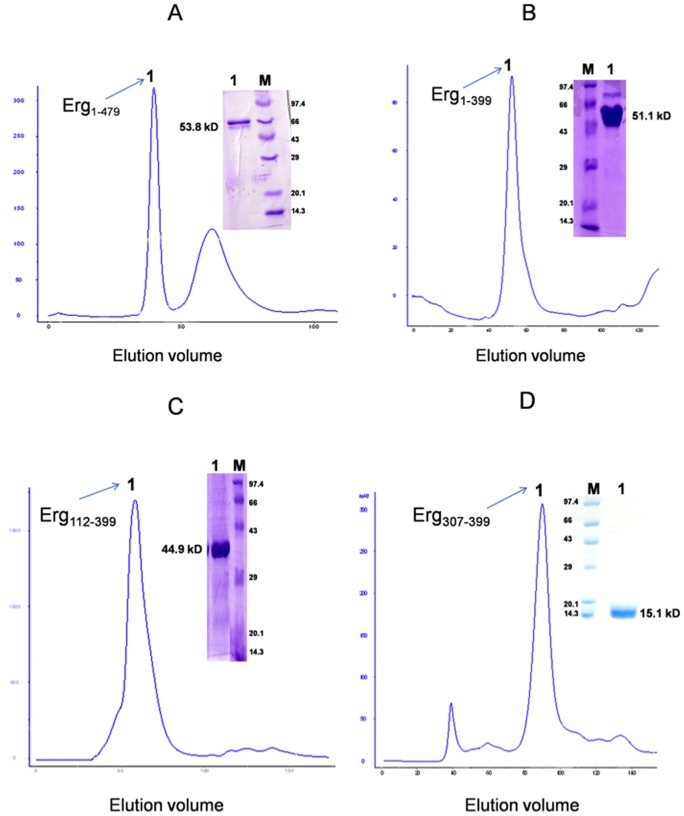
Size exclusion chromatography of Ergp55 polypeptides. The chromatogram of (A) full length Erg_1–479_ (B) Erg_1–399_ (C) Erg_112–399_ and (D) Erg_307–399_ polypeptides are shown. The SDS-PAGE analysis and calculated molecular weight of each protein are denoted on each chromatogram.

N-terminal protein sequencing and ion spray mass spectrometry confirmed identity and purity of Ergp55 polypeptides. Protein concentration was determined using absorbance at 280 nm. Coomassie brilliant blue stained SDS-PAGE analysis indicated that all Ergp55 polypeptides were purified greater than 95% purity. All proteins were stored at −20°C.

### Surface plasmon resonance experiment

Biosensor studies were performed using BIAcore 2000 (Biacore Pharmacia Biosensor AB, Uppsala Sweeden) equipment [18]. All experiments were performed at 25°C in HBS-buffer containing [10 mM HEPES buffer pH 7.4, 150 mM NaCl, 50 mM EDTA, 0.005% P20 as surfactant). Since all Ergp55 polypeptides contain 6xHis tag either on N- or C-terminal, the sensor chip containing high density of immobilized Ni-NTA is an ideal tag to immobilize these proteins. Initially, the flow cells of the chip were activated by passing nickel chloride solution over it. 40 ul of each Ergp55 polypeptide *e.g*., Erg_1–479_ (0.14 ug/ul), Erg_1–399_ (0.19 ug/ul), Erg_112–399_ (0.92 ug/ul) and Erg_307–399_ (0.17 ug/ul) were injected in different flow cells of Ni-NTA chip at flow rate of 10 ul/min.

**Figure 3:**
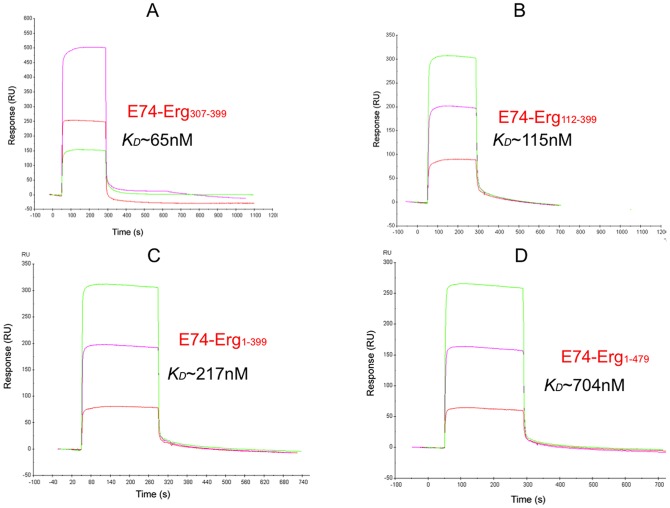
Binding analysis of DNA sequence of E74 promoter to immobilized Ergp55 polypeptides. In figures (A) Erg_1–479_ (B) Erg_1–399_ (C) Erg_112–399_ and (D) Erg_307–399_ polypeptides were immobilized on Ni-NTA chip. Three concentration (1, 2, 3 μM) of DNA sequence of E74 promoter injected on each immobilized Ergp55 polypeptide.

In different flow cells of Ni-NTA chip, following RU unit of each Ergp55 polypeptide was immobilized *e.g*., Erg_1–479_ (2904 RU), Erg_1–399_ (2848 RU), Erg_112–399_ (2825 RU) and Erg_307–399_ (247 RU), where 1 RU corresponds to immobilized protein of concentration ∼1 pg/mm. Binding experiments were performed with three different concentration [1, 2, 3 μM) of DNA sequence of E74 promoter (5′ TACCGGAAGT 3′) in HBS-buffer and injected over immobilized Ergp55 polypeptides at flow rate of 10 μl/min. Similar experiment was performed using DNA sequence of cfos promoter sequence (5′ GACAGGATGTG 3′). The sensogram allowed to run for another 4 min. The regeneration of biosensor surface was done using 30 s pulse of 1 mM NaOH at flow rate of 10 μl/min. Associate and dissociation kinetic constants were calculated by BIAeveluation 3.0 software using simple 1∶1 Langmuir model with the assumption, that density of Ergp55 polypeptides on the sensor chip were not high enough to support bivalent DNA binding.

**Figure 4:**
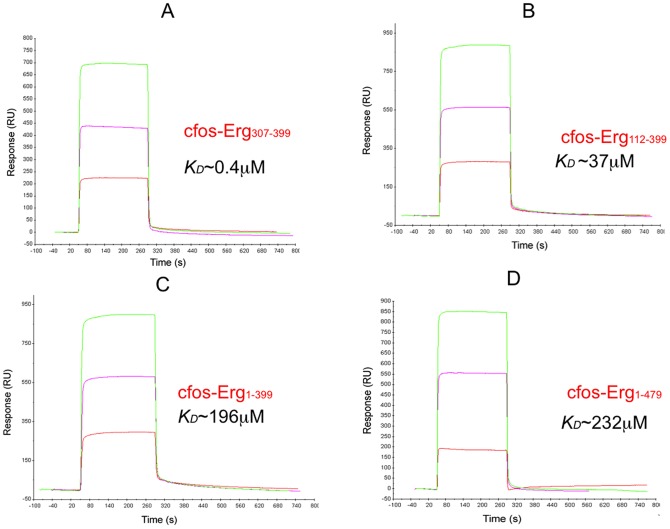
Binding analysis of DNA sequence of cfos promoter to immobilized Ergp55 polypeptides. In figures (A) Erg_1–479_ (B) Erg_1–399_ (C) Erg_112–399_ and (D) Erg_307–399_ polypeptides are immobilized on Ni-NTA chip. Three different concentration (1, 2, 3 μM) of DNA sequence of cfos promoter injected on each immobilized Ergp55 polypeptide.

### Circular dichorism

CD measurements were recorded using Chirascan^TM^ CD spectropolarimeter (Applied Photophysics) with a water bath to maintain the constant temperature. The Ergp55 polypeptides were diluted to 0.2 mg/ml in 10 mM sodium phosphate buffer, pH 8.0 and loaded on 0.1 cm quartz cuvette. The blank of all experiments was 10 mM sodium phosphate buffer, pH 8.0. The final spectrum was an average of three sequential scan.

**Figure 5:**
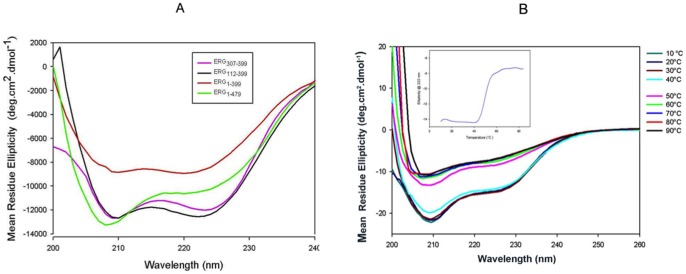
CD spectra of Ergp55 polypeptides. (A) CD spectra of Erg_1–479_, Erg_1–399_, Erg_112–399_ and Erg_307–399_ polypeptides recorded from 200 nm to 260 nm. (B) Temperature induced unfolding of full length Ergp55 protein. The plot (inner) containing mean residue ellipticity versus temperature indicates the denaturation of α-helices of folded full length Ergp55.

For thermal denaturation study of full length Ergp55, the CD spectra were recorded at 10°C increment starting from 10°C to 90°C. Before measurement, the sample cuvette was equilibrated at each temperature. Temperature readings were taken within cuvette holder agreed with temperature of water bath. All CD data were converted to mean residue ellipticity (deg. cm^2^/dmol). The Dichroweb server [19] was used to estimate the amount of secondary structure in Ergp55 polypeptides from CD spectra.

### Secondary structure prediction

The SOPMA [20], GOR [21] and PSIPRED [22] algorithms were used to predict the secondary structure contents in full length Ergp55, which showed ∼21% α-helix, 12–15% β-sheet and 65–69% random coil structures.

**Figure 6:**
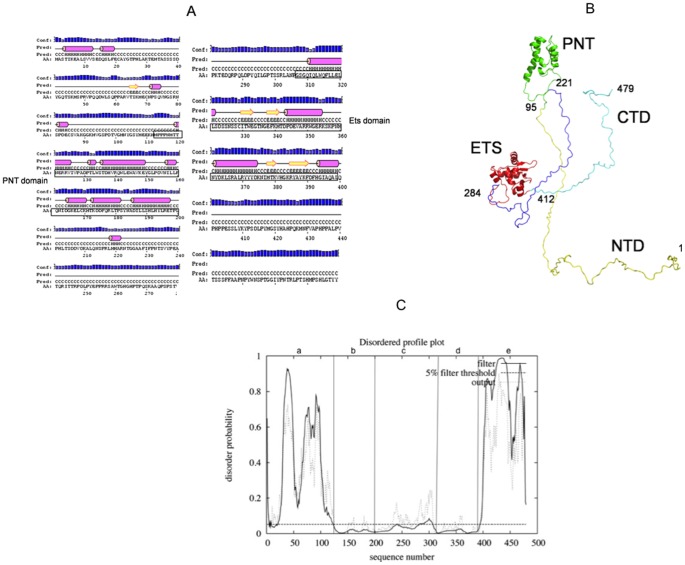
Model of Ergp55 polypeptides. (A) PSIPRED program analysis of full length Ergp55. (B) Structural model of full length Ergp55 after molecular modeling and dynamics simulation analysis using GROMACS program (C) The disordered prediction made by DISOPRED for (a), NTD (b), PNT (c), CAE/CD (d), ETS and (e), CTD regions of Ergp55.

### Modeling of Ergp55 polypeptides

Phyre server [23] was used to obtain the structure of full length Ergp55 protein (1–479 residues). The server yielded the structure of PNT domain (95–221 residues) of Ergp55 using template PDB-2YTU (solution structure of SAM-PNT domain of human friend leukemia integration factor-1 transcription factor, not yet published). The NMR solution structure of PNT domain (108–201 residues) was obtained from protein data bank (PDB-1SVO) [24]. The Phyre server also yielded the structure of Ets domain (284–412 residues) of Ergp55 using the template PDB-2NNY (Regulation of transcription factor Ets-1 by DNA mediated homodimerization) [13]. The NMR structure of Ets domain of Fli1 (306–403 residues) was obtained from protein data bank (PDB-1FliA) [25]. The structural modeling of N- terminal (1–94 residues), central domain (222–283 residues) and C- terminal (413–479 residues) of Ergp55 were not attempted due to lack of input model.

The Modeler [26] and LOMETS threading [27] programs were used to build the structure of full length Ergp55 using following inputs (i) structural model of PNT domain (95–221 residues) of Ergp55 (ii) structural model of Ets domain (284–412 residues) of Ergp55 (iii) NMR structure of PNT domain (108–201 residues) of Ergp55 and (iv) NMR structure of Ets domain (306–403 residues) of Fli1. Energy minimization was performed on modeled Ergp55 using Gromacs program version 4.0.5 [28]. 100 steps of steepest decent and 500 steps of conjugated gradient algorithms were used in energy minimization calculation.

### Molecular dynamics simulations

The 10 ns molecular dynamics (MD) simulation was performed on minimized Ergp55 model using GROMACS program (version 4.0.5) with Gromacs43a2 force field [28]. The Ergp55 model was immersed in a cubic box extending 0.5 nm from the protein surface and solvated with explicit SPC water molecules. Chloride and sodium ions were added to neutralize the systems, which were then simulated with periodic boundary conditions. The solvated Ergp55 model consists of 4832 protein atoms surrounded by ∼390, 000 water molecules. Before running the simulation, whole system was energy minimized for 200 iterations of steepest descents and then equilibrated for 20 ps keeping protein atoms restrained. All restraints were removed from the protein and temperature was gradually increased in 10 distinct steps of 5 ps simulations each.

Berendsen coupling was employed to maintain a constant temperature of 300 K with a coupling constant τ of 0.1 ps. Van der Waals interactions were modeled using 6–12 Lennard-Jones potentials with 1 nm cutoff. The coulomb cut off was 1.0. The time step employed was 2 fs and coordinates were saved every 5 ps for analysis of MD trajectories.

The stereochemistry of simulated Ergp55 model was checked by PROCHECK program of CCP4 suite [29]. Secondary structure composition was measured by DSSP program [30] and structure visualization by PyMOL program [31].

## Results

### Purification of recombinant Ergp55 polypeptides

We produced full length and smaller polypeptides (containing subset of predicted domains) of Ergp55 in *E. coli* and purified using standard chromatographic techniques (Fig. 1A–B). During size exclusion chromatography, the purified Erg_1–479_, Erg_1–399_, Erg_112–399_ and Erg_307–399_ polypeptides eluted at volumes corresponding to their molecular weights (Fig. 2A–D). The full length Erg_1–479_ eluted at the size of 53.8 kD, Erg_1–399_ polypeptide eluted at 51.1 kD, Erg_112–399_ eluted at 44.9 kD and Erg_112–399_ eluted at 15.1 kD. The Erg_1–479_ and Erg_1–399_ polypeptides have tendency to degrade if kept for 7 days at 4°C. The Erg_112–399_ and Erg_307–399_ polypeptides do not degrade over time and more stable than Erg_1–479_ and Erg_1–399_ polypeptides.

### DNA binding studies using E74 and cfos promoter sequences

The functionality of purified Ergp55 polypeptides was assessed by DNA binding experiment using surface plasmon resonance technique. The observed *K_D_* value of Ergp55 polypeptides with DNA sequence of E74 promoter were *e.g*., Erg_1–479_ ∼ 704 nM, Erg_1–399_ ∼ 217 nM, Erg_112–399_ ∼ 115 nM and Erg_307–399_ ∼ 65 nM (Fig. 3A–D). These results indicated that Ets domain of Ergp55 (Erg_307–399_) has the highest affinity for E74 promoter DNA sequence. The DNA binding affinity decreases ∼ 2 fold for Erg_112–399_ polypeptide, ∼3 fold for Erg_1–399_ polypeptide and ∼10 fold for full-length Erg_1–479_, when compared with Erg_307–399_ polypeptide (Ets domain). These results indicate that N- as well as C- terminal domains with respect to Ets domain are involved in auto-inhibition of DNA binding to Ergp55 protein.

The observed *K_D_* value of Ergp55 polypeptides with cfos promoter sequence were *e.g*., Erg_1–479_ ∼ 232 μM, Erg_1–399_ ∼ 196 μM, Erg_112–399_ ∼ 38 μM and Erg_307–399_ ∼ 0.45 μM (Fig. 4A–D). These results also indicate that Ets domain has the highest affinity for DNA sequences of cfos promoter. Both N- and C- terminal domains with respect to Ets domain are involved in inhibition of cfos DNA binding to Ergp55 protein.

### CD measurements of Ergp55 polypeptides

To identify the secondary structure contents in Ergp55 polypeptides, the far-UV CD spectroscopy was used (Fig. 5A). The CD data were de-convoluted using DICHROWEB web server [19] and percentage of α-helix, β-sheet and random coil structures were estimated. The CD spectra of full-length Erg_1–479_ (Fig. 5A) showed two minima around 208 nm and 222 nm, a characteristic of α-helical structure. Deconvolution of data predicts ∼35% α-helix, 15% β-sheet and 49% random coil structures in full length Ergp55. The CD data of Erg_1–399_ polypeptide predicts ∼25% α-helix, 17% β-sheet and 57% random coil structures, which shows less α-helix and β-sheet structure compare to full length Erg_1–479_ structure.

In case of Erg_112–399_ polypeptide, the CD data estimates ∼29% α-helix, 15% β-sheet and 55% random coil structures. This polypeptide contains less α-helix, similar β-sheet structure compared to full length Erg_1–479_. For Erg_307–399_ polypeptide, the CD data estimates ∼31% α-helix, 10% β-sheet and 59% random coil structures, which has less α-helix and β-sheet structure compare to full length Erg_1–479_ structure. However, these values are close to secondary structure contents in crystal structure of Ets domain of Fli-1 (35.7% α-helix, 4.1% β-sheet, 60.2% random coil). The Ets domain of Fli-1 is the closest homologous to Ets domain of Ergp55 [32].

### Thermostability of full length Ergp55

To assess the thermostability of full length Ergp55, a far UV-CD spectrum of protein was measured from 10 to 90°C (Fig. 5B). It is clear from spectra that secondary structure of Ergp55 denatures as temperature increased. When mean residue ellipticity at 222 nm is plotted against temperature, the inflection point of sigmoidal curve indicates the T_m_ of 45±2°C of full length Ergp55.

### Molecular modeling and dynamic simulation of full length Ergp55

The secondary structure prediction on full length Ergp55 using PSIPRED program is shown in (Fig. 6A). The Modeler and automatic threading LOMETS programs were used to construct full length Ergp55 model using (i) structure of 95–221 residues of Ergp55 using the template PDB-2YTU (not published) having 57% sequence identity (ii) the structure of 284–412 residues of Ergp55 using template PDB-2NNY [13] having 40% sequence identity and (iii) NMR structure of 108–201 residues of Ergp55 and (iv) NMR structure of 306–403 residues of Ets domain of Fli-1 having 90% sequence identity. Energy minimization and dynamics simulations analysis were performed on constructed Ergp55 model, which yielded a flexible and elongated structure (Fig. 6B). The Ergp55 structure remained very stable during whole simulation time, as confirmed by all the indicators commonly used to analyze MD simulation.

The 93% residues of Egp55 model lie in most favored region of Ramachandran plot and a Prosa Z-score of −4.87. DISOPRED [33] analysis on Ergp55 model indicated that N-terminal (1–118 residues) and C-terminal (397–479 residues) are largely disordered (except N-terminal 4–23 residues) and remaining Ergp55 structure (119–396 residues) were ordered (Fig. 6C). The structured PNT domain contains tertiary arrangement of four α-helices, characteristic of large group of SAM domain [34]. The Ets domain consists of four α-helices and fourβ-sheets, a characteristic of Ets family proteins. In N-terminal domain, 15 residue stretch predicted to form α-helix and 3 residue long helix (219–221 residues) are observed in in CAE/CD domain of Ergp55. The stretches of residues in C-terminal domain of Ergp55 predicted to have only random coil structure.

The N- terminal and C-terminal domain of Ergp55 are positioned away from Ets domain. The DNA binding groove of Ets domain is exposed to solvent and free to bind promoter DNA sequences. The CAE/CD domain is positioned between PNT and Ets domain to regulate the activity of Ergp55. The N- terminal and C-terminal domains of Ergp55 are positioned in a region that do not prevent the DNA binding activity of Ets domain and play a role in transcriptional activation and localization of Ergp55.

## Discussion

In current study, we have expressed full length and smaller polypeptides of Ergp55 in *E. coli*. The combinations of two chromatography steps (Ni-NTA affinity and size exclusion chromatography) have yielded more than 95% pure Ergp55 polypeptides based on mass spectrometry and SDS-PAGE analysis. Prior to structural studies, the activity of purified Ergp55 polypeptides were checked by binding studies using DNA sequences of E74 and cfos promoters. The surface plasmon resonance technique was used for binding analysis. These results indicated that Ergp55 polypeptides produced in *E. coli* were in good conformation and bind specifically DNA sequences of E74 and cFos promoters with different affinities.

### 

#### DNA binding autoinhibition of Ergp55

In case of E74 promoter sequence, following *K_D_* values were observed for Ergp55 polypeptides (i) Erg_307–399,_ 65 nM (ii) Erg_112–399,_ 115 nM (iii) Erg_1–399_, 217 nM and (iv) full length Erg_1–479_, 704 nM. Comparison of (i) and (ii) indicated that N-terminal region (PNT+CAE/CD domains) preceding to Ets domain inhibit the E74 DNA binding to Ets domain. Comparison of (ii) and (iii) showed the evidence of increased DNA binding inhibition by having NTD domain in Erg_1–399_ polypeptide. Comparison of (iii) and (iv) indicate that adding CTD domain in Erg_1–399_ polypeptide showed enhanced inhibition in DNA binding to Ets domain. These results indicate that E74 DNA binding to Ergp55 is negatively influenced by CAE/CD, PNT and NTD domains located at N-terminal and CTD domain located C-terminal region in Ergp55.

With cfos promoter DNA sequence, following *K_D_* values were obtained for Ergp55 polypeptides (i) Erg_307–399,_ 0.4 μM (ii) Erg_112–399,_ 37 μM (iii) Erg_1–399_, 196 μM and (iv) full length Erg_1–479_, 232 μM. These results indicate that cfos promoter sequence bind with different affinity to Ergp55 polypeptides than E74 promoter sequence, however mechanism of DNA binding inhibition was similar as observed in case of E74 promoter DNA sequence.

A cooperatively acting DNA inhibiting region (468–510 residues) was identified at C-terminal of Ets-1 transcription factor [34]. In case of ERM and PEA3 transcription factors, two main domains located at N- and C-terminal with respect to their ETS- domain inhibiting DNA binding affinity [35]–[38]. One domain corresponds to residue 280–360 residues of ERM transcription factor, involved in inhibition of ERM DNA binding capacity. These domains are rich in proline residues generally devoid α-helical structures. The mechanism by which two domains cooperatively inhibit ERM DNA binding is different than observed in case of Ets-1 transcription factor. The DNA binding activity of Ets domain is dependent on autoinhibitory module [39]. The binding affinity of Ets domain Ergp55 to E74 and cfos promoter DNA was consistent to the observation obtained in case of above transcription factors.

#### Circular dichorism analysis of Ergp55 polypeptides

The circular dichorism technique was used to identify the secondary and tertiary structures of Ergp55 polypeptides. The full length and smaller Ergp55 polypeptides contain high α-helical and random coil structures. The CD data estimates 35% α-helix and 49% random coil structures in full length Ergp55 protein. Examinations of thermal stability and temperature effect on full length Ergp55 protein indicated that protein underwent an alteration of secondary structure upon heating. The secondary structure is regained after cooling the protein from 80°C to 20°C. Short change of temperature is unlikely to have any effect on secondary structure of Ergp55 protein.

#### Modeling and dynamics simulation of full length Ergp55

The molecular modeling and dynamics simulation analysis indicated that full length Ergp55 acquires a flexible and highly elongated structure. Only PNT and Ets domains are structured in protein and long flexible regions are observed at N- and C- terminus of Ergp55. The structure of PNT domain of Ergp55 consists of four-helix bundle Sam like structure (34). The Ets domain of Ergp55 is structured in a winged helix-turn-helix with scheme α_1_β_1_β_2_α_2_α_3_β_3_β_4_α_4_ [40]–[41]. The central CAE/CD domains contain one small helix at position 220. The secondary and disordered prediction analysis on Ergp55 also supported the finding observed in modeling and dynamics simulation studies of Ergp55. All these observations supported the flexible, non-globularity and highly elongated structure of Ergp55 protein.

In conclusion, we have characterized the recombinant full length and smaller polypeptides of Ergp55 produced in *E. coli*. The Ergp55 polypeptides were purified greater than 95% purity as determined by mass spectrometry and SDS-PAGE analysis. The structural data presented here showed the evidence of flexible and highly elongated structure of full length Ergp55 protein. The binding analysis using DNA sequences of E74 and cfos promoters indicate that longer fragments of Ergp55 (beyond the canonical Ets domain) showed the evidence of autoinhibition.
